# Is a HIV vaccine a viable option and at what price? An economic evaluation of adding HIV vaccination into existing prevention programs in Thailand

**DOI:** 10.1186/1471-2458-11-534

**Published:** 2011-07-05

**Authors:** Pattara Leelahavarong, Yot Teerawattananon, Pitsaphun Werayingyong, Chutima Akaleephan, Nakorn Premsri, Chawetsan Namwat, Wiwat Peerapatanapokin, Viroj Tangcharoensathien

**Affiliations:** 1 Health Intervention and Technology Assessment Program (HITAP), 6th Floor, 6th Building, Department of Health, Ministry of Public Health, Tiwanon Rd., Amphur Muang, Nonthaburi, Thailand; 2 International Health Policy Program (IHPP), Ministry of Public Health, Tiwanon Rd., Amphur Muang, Nonthaburi, Thailand; 3 Department of Disease Control, Ministry of Public Health, Tiwanon Rd., Amphur Muang, Nonthaburi, Thailand; 4 East-West Center, Hawaii, U.S.A./Policy Research Development Institute Foundation (PRI), Tiwanon Rd., Amphur Muang, Nonthaburi, Thailand

**Keywords:** Vaccine, HIV, AIDS, Economic evaluation, Cost-utility analysis

## Abstract

**Background:**

This study aims to determine the maximum price at which HIV vaccination is cost-effective in the Thai healthcare setting. It also aims to identify the relative importance of vaccine characteristics and risk behavior changes among vaccine recipients to determine how they affect this cost-effectiveness.

**Methods:**

A semi-Markov model was developed to estimate the costs and health outcomes of HIV prevention programs combined with HIV vaccination in comparison to the existing HIV prevention programs without vaccination. The estimation was based on a lifetime horizon period (99 years) and used the government perspective. The analysis focused on both the general population and specific high-risk population groups. The maximum price of cost-effective vaccination was defined by using threshold analysis; one-way and probabilistic sensitivity analyses were performed. The study employed an expected value of perfect information (EVPI) analysis to determine the relative importance of parameters and to prioritize future studies.

**Results:**

The most expensive HIV vaccination which is cost-effective when given to the general population was 12,000 Thai baht (US$1 = 34 Thai baht in 2009). This vaccination came with 70% vaccine efficacy and lifetime protection as long as risk behavior was unchanged post-vaccination. The vaccine would be considered cost-ineffective at any price if it demonstrated low efficacy (30%) and if post-vaccination risk behavior increased by 10% or more, especially among the high-risk population groups. The incremental cost-effectiveness ratios were the most sensitive to change in post-vaccination risk behavior, followed by vaccine efficacy and duration of protection. The EVPI indicated the need to quantify vaccine efficacy, changed post-vaccination risk behavior, and the costs of vaccination programs.

**Conclusions:**

The approach used in this study differentiated it from other economic evaluations and can be applied for the economic evaluation of other health interventions not available in healthcare systems. This study is important not only for researchers conducting future HIV vaccine research but also for policy decision makers who, in the future, will consider vaccine adoption.

## Background

HIV infection leading to AIDS has become the most serious cause of disability adjusted life years lost in the Thai population [1]. Thailand is often referred to as a success story because of the fact that it has slowed down the HIV epidemic through several effective measures, including public education campaigns, the prevention of mother-to-child transmission (PMTCT) through the use of antiretroviral drugs and formula feeding, a 100% condom use program among commercial sex workers, etc. [2-5]. However, high HIV prevalence has recently been observed among particular population groups: men who have sex with men (MSM), injecting drug users (IDUs), female sex workers (FSWs), and migrant workers. In 2010, a total of 532,500 people were living with HIV and 12,800 new infections were reported in Thailand [6].

Because the above HIV prevention interventions proved that they had only a limited effect on the spread of HIV, the development of new preventive tools remains important, especially for some hard-to-reach population groups. Previous published economic evaluations of HIV vaccination have been conducted in many settings, including Thailand, and have shown promising results in terms of restraining the spread of the disease among the general population [7], women [8], new-borns [9], and children aged up to 10 years old [10]. A randomized clinical trial on the prime-boost combination of HIV vaccination (ALVAC-HIV^® ^and AIDVAX B/E^®^) was established in Thailand to evaluate the vaccination program's efficacy among 16,402 members of the general population aged between 18 and 30 years old [11]. The trial was conducted by the Ministry of Public Health-Thai AIDS Vaccine Evaluation Group (MOPH-TAVEG) under the conditions of a prior agreement. If the vaccine shows promising results and it can be registered in the Thai market, the company will give special privileges to the government to purchase the vaccine at a discounted price.

As a result, prior to the announcement of the trial results in September 2009, this economic evaluation was conducted at the request of policy makers aiming to determine policy decisions regarding the adoption and price negotiation of the vaccine. This study aims to determine the maximum price at which the vaccine remains cost-effective in the Thai healthcare setting. It also aims to identify the relative importance of vaccine characteristics, i.e. cost of vaccination, vaccine efficacy, duration of protection, and vaccine acceptance rates, as well as risk behaviors that have changed post-vaccination, all of which may affect the vaccine's cost-effectiveness.

## Methods

### Study design

**Figure 1:**
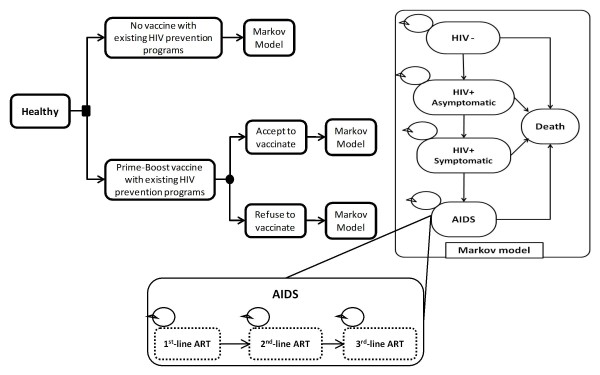
**A semi-Markov model**. The provision of HIV vaccination was on a voluntary basis, so the target population had the option of accepting or refusing the vaccine. The model consists of five health states: (1) HIV negative; (2) HIV positive without symptoms (asymptomatic); (3) progression into HIV with related symptoms (symptomatic); (4) AIDS state during antiretroviral treatment (ART), which includes three sub states, i.e., the first-line ART, the second-line ART, and the third-line ART regimens; and (5) death due to HIV infection and other causes. The arrows represent the probability of transitions from one state to another.

This is a model-based economic evaluation for which a semi-Markov model (Figure 1) was developed using Microsoft excel^® ^(Microsoft Corp., Redmond, WA) in order to estimate the incremental cost-effectiveness ratio (ICER) in terms of cost per quality-adjusted life year (QALY). The HIV vaccination combined with the existing HIV prevention programs was compared to the existing HIV prevention programs without HIV vaccination. The assessment was made from a government perspective.

The existing HIV prevention programs include 1) a condom use program, which provides free condoms in readily visible and accessible sites through healthcare facilities and private businesses serving population groups at high risk of sexually transmitted diseases (STDs) and HIV; 2) school-based education, which provides information to young people and reinforces healthy norms in a school setting; 3) anti-retroviral prophylaxis for vertical HIV transmission, which delivers free voluntary HIV counseling and testing (VCT) services for all pregnant women, as well as providing HIV infected pregnant women with free antiretroviral treatment (ART), breast milk substitutes for 12 months, and counseling with their partner to test their newborn babies at 12 and 18 months and recruit them into universal ART programs when CD4 counts indicate the necessity; 4) diagnosis and treatment of sexually transmitted infections; 5) a needle and syringe program, which offers the opportunity for IDUs who continue injecting to safely dispose of used needles and syringes and to obtain clean drug injection equipment at no cost; and 6) screening blood products and donated organs for HIV.

The analysis focused on particular target population groups: the general population aged 18 to 30 years old, FSW, IDU, MSM, and military conscripts. As mentioned already, at the time this study was conducted, there was no HIV vaccine available in the market, nor did the results of the HIV vaccine efficacy trial in Thailand reveal anything conclusive [11]. A number of assumptions relating to population behavior patterns and vaccine characteristics have been put forward as follows:

(1) Vaccine efficacy: A figure of 50% vaccine efficacy was applied for the base-case scenario, and a range of 30%-70% was used in the uncertainty analysis.

(2) Duration of protection: It was assumed in the base-case scenario that the vaccine's effects last for 10 years; however, both longer and shorter time periods of vaccine protection were used in the uncertainty analysis.

(3) Vaccine cost: The base case analysis applies a vaccine cost of 3,500 Thai baht (THB), or approximately US$100 (US$1=34 THB in 2009), as recommended by the International AIDS Vaccine Initiative [12] for a full course of immunization (6 doses each of prime and boost injections). The study varies significantly regarding the vaccine cost when examining the maximum level at which the vaccine still offers viable cost-effectiveness [12,13].

(4) Vaccine acceptance rates: Research by Suraratdecha et al. [14] reported that nearly 80% of adults would accept HIV vaccination if it were free of charge. Therefore, the base-case scenario adopts an 80% acceptance rate, while a range of 30% to 100% was applied in the uncertainty analysis.

(5) Change in risk behaviors as a result of receiving the vaccine: This study assumed change of risk behaviors in terms of the decreasing rate of condom use for all target population groups and an increasing rate of needle sharing for IDUs from 0% (unchanged) to 30% (significant change) [15,16].

In addition, the model was run to predict relevant costs and outcomes over a 99-year period (lifetime horizon). Both future costs and outcomes were discounted at the rate of 3% per annum as suggested by the Thai economic evaluation guideline [17].

### Epidemiological data

#### Baseline HIV incidence

The age-specific incidences of HIV infection among the general population in Thailand were used to estimate the probability of moving from a HIV negative state to an HIV asymptomatic state (early HIV positive) [18]. For four high-risk population groups, statistics for the annual incidences of HIV infection were obtained from the Sentinel Sero-Surveillance survey conducted at the national level by the Bureau of Epidemiology, the Ministry of Public Health (MoPH), and some cohort studies [19-21]. In addition, the average duration that FSWs, IDUs, MSM, and male military conscripts remain at high-risk is 4, 10, 20, and 2 years, respectively [6]. It was presumed that after these periods, high-risk groups would change their behavior, and that the annual probability of contracting HIV would then be similar to that of the general population.

#### HIV/AIDS progression

The annual probabilities of progressing from being HIV asymptomatic to HIV symptomatic among Thai people living with HIV/AIDS was identified from published literature [22]. The progression from an HIV symptomatic state to an AIDS state was calculated from the ratio between the number of new AIDS patients at the end of 2008 and the number of people living with HIV in that year [6,10]. The AIDS patients in this study were assumed to have started the first-line ART regimen and then switched to the second-line regimen and onto the third-line regimen. It was noted that the third stage may cause patients to develop severe adverse effects, drug resistance, or major opportunistic infections. This information was obtained from an HIV cohort consisting of 646 patients receiving ART [23].

**Figure 2:**
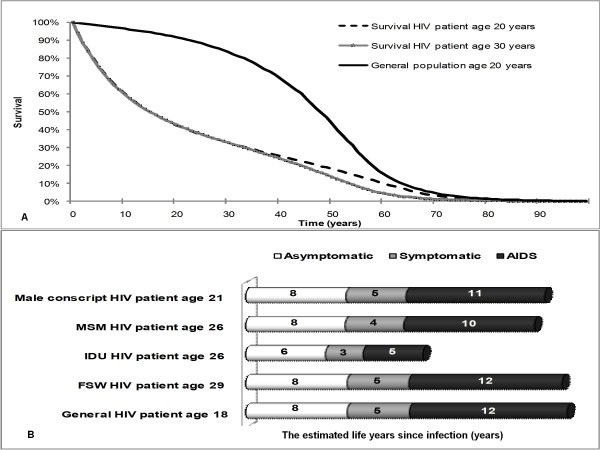
**Model validation**. (A) The survival curve of HIV patients compared to the general population (B) The estimated life years since infection classified by risk group MSM: men who have sex with men; IDU: injecting drug user; and FSW: female sex worker

The annual mortality rates among HIV asymptomatic, HIV symptomatic and AIDS patients were estimated from two cohort studies in Thailand. The two cohorts were made up of 880 HIV/AIDS patients [23,24]. The age-adjusted annual probability of dying from other causes was derived from the Burden of Disease and Injury Study in Thailand [1]. The epidemiological data derived from the model, i.e. the survival of HIV patients compared to the general population and the average duration of each health state (Figure 2), were finally approved by Thai HIV experts in a meeting held on 18 September, 2009.

#### Change in risk behaviors

This analysis also assumed an increase in risk behavior among those vaccinated, i.e. decreased condom use among the general population, FSWs, MSM, and male military conscripts, as well as increased needle sharing among IDUs. It is expected that the increased risk behavior would range from slight (10%) to significant (30%). The impact of risk behavioral changes post-vaccination was estimated by using a dynamic model: the "Asian Epidemic Model (AEM)" (Wiwat Peerapatanapokin, East-West Center, Hawaii, U.S.A./Policy Research Development Institute Foundation (PRI), personal communication, September 29, 2009). The AEM is a simulation model that simulates behavior, dynamics linkages, and interaction among subpopulations. It has been officially used for HIV/AIDS projection in Thailand since the year 2000 [6].

### Costs data

All costs were converted into and reported in 2009 THB rates [25]. For international comparison, costs were converted into international dollars using a purchasing power parity (PPP) exchange rate (a PPP 2009 dollar = 16.55 THB) [26]. Based on the government perspective, only direct medical costs were included. The National AIDS Spending Assessment in Thailand reported the total national expenditure on HIV prevention; the figures for the target population were derived from the Department of Local Administration, while the Ministry of the Interior predicted the cost of HIV prevention per target person [27,28].

The costs associated with HIV vaccination included vaccine costs (i.e. price per dose, delivery costs, and storage costs), costs of community engagement, and costs of pre- and post-HIV vaccination counseling. Also, the study assumed that each booster revaccination required a full course and that revaccination was needed in order to gain effective protection over a 30-year time period, which was thought to cover the period when people are at high risk of infection. Community engagement is needed to raise awareness of the vaccine among the target population, and this would also help to obtain a high vaccine acceptance rate. The cost of 937 THB per individual form the target population was estimated in a vaccine clinical study in Thailand (Nakorn Premsri, National Vaccine Committee Office, Department of Disease Control, MoPH, personal communication, August 25, 2009).

The annual direct medical costs of HIV/AIDS treatment, including the costs of ART, were obtained from the National Health Security Office (NHSO). The costs of the three ART regimens were derived from the National database (Thanapat Laowahutanon, AIDS office, Bureau of Disease Management, NHSO, personal communication, August 20, 2009) and adjusted to match the proportion of utilization among patients, which changes over time as a result of ART resistance. The costs of laboratory testing, follow-up treatment, and treatment of opportunistic infections were derived from a study entitled "The economics of effective AIDS treatment study in Thailand" [29].

### Health outcome variables

QALY was used as an outcome measure to make a comparison between HIV vaccination programs and existing prevention programs. Utility weights (0 = death and 1 = full health) for calculating QALY were obtained from Leelukkanaveera's study [24], which collected the quality of life data of 1,277 HIV infected patients in 16 community hospitals in Thailand using the EQ-5D instrument. The HIV/AIDS patients' quality of life was classified by disease stage, i.e. asymptomatic HIV, symptomatic HIV, and AIDS, to be 0.86, 0.80, and 0.76, respectively.

### Uncertainty analysis

One-way sensitivity analysis was performed to examine the relative importance of the four parameters assumed. These are vaccine efficacy, duration of vaccine protection, vaccine acceptance rate, and change in risk behavior. Only significant parameters identified from one-way sensitivity analysis were used in the threshold analysis to quantify the maximum costs of the vaccine to the given ceiling threshold of 100,000 THB per QALY gained [30]. Based on the statement of the Subcommittee for the Development of the National List of Essential Drugs and the Subcommittee for the Development of the Health Benefit Package and Service Delivery of the NHSO in 2007, the societal willingness to pay (WTP) threshold for a QALY gained for the adoption of health interventions is 100,000 THB (6,000 PPP$), approximating per capita Gross Domestic Product (GDP) [30]. This value is in accordance with the recent community survey aimed at identifying households' willingness-to-pay for a QALY, the detailed approach of which will be published elsewhere [31].

In addition, probabilistic sensitivity analysis (PSA) was conducted to assess uncertainty surrounding all model parameters given the mean, standard error (SE), and distribution of each parameter as shown in Table 1, 2 and 3. Probability distributions were defined as follows [32]: (1) beta-distributions were assigned where parameter values ranged from zero to one, such as transition probabilities and utility parameters; (2) gamma-distributions were specified when parameter values were above zero and positively skewed by costs variables; and (3) a log-normal distribution was used for survival parameters. The PSA was conducted by using a second order Monte Carlo simulation performed by Microsoft excel^® ^(Microsoft Corp., Redmond, WA) and run for 1,000 iterations to yield a range of plausible values for lifetime costs, quality-adjusted life years (QALYs), and incremental cost-effectiveness ratios (ICERs) [33]. The results were depicted in graphs that plotted the probability of HIV vaccination being cost-effective against different vaccine costs.

**Table 1:** Input parameters (i.e. discount rate and transition probabilities) used in Markov model

Parameters	Distribution	Mean	SE	References
Yearly discount rate (%)				
Costs (range)		3 (0-6)		[17]
Outcome (range)		3 (0-6)		[17]
Transition probabilities				
*Probabilities of HIV infection classified by risk group*				
Annual incidences of HIV infected in general population aged 18 years	Beta	0.001		[18]
Annual incidences of HIV infection in FSW	Beta	0.022	0.016	[19]
Annual incidences of HIV infection in IDU	Beta	0.034	0.002	[20]
Annual incidences of HIV infection in MSM	Beta	0.055	0.010	[21]
Annual incidences of HIV infection in male conscripts	Beta	0.002	0.001	[19]
*Transition probabilities of HIV positive with asymptomatic state*				
Annual progression risk from asymptomatic to symptomatic state	Beta	0.865	0.047	[46]
Annual death risk of asymptomatic state	Beta	0.058	0.008	[22]
*Transition probabilities of HIV positive with symptomatic state*				
Annual probability to progress from HIV to AIDS	Beta	0.087	0.0004	[6,10]
Constant in survival analysis for baseline hazard	Lognormal	-8.38	1.44	[24]
CD4 coefficient in survival analysis for baseline hazard	Lognormal	-0.01	0.001	[24]
Ancillary parameter in Weibull distribution	Lognormal	0.04	0.19	[24]
Average CD4 of patients (#patients=234)	Lognormal	321.44	9.46	[24]
*Transition probabilities of AIDS state to death*				
Constant in survival analysis for baseline hazard	Lognormal	-4.81	0.86	[23]
Age coefficient in survival analysis for baseline hazard	Lognormal	-0.04	0.02	[23]
CD4 coefficient in survival analysis for baseline hazard	Lognormal	-0.02	0.00	[23]
Ancillary parameter in Weibull distribution	Lognormal	-0.33	0.11	[23]
Average CD4 of patients (#patients=646)	Gamma	81.01	2.67	[23]
*Transition probability of switching from first-line to second-line ART regimen*				
Constant in survival analysis for baseline hazard	Lognormal	-6.17	0.52	[23]
CD4base coefficient in survival analysis for baseline hazard	Lognormal	0.003	0.001	[23]
Age coefficient in survival analysis for baseline hazard	Lognormal	0.0313	0.0113	[23]
Ancillary parameter in Weibull distribution	Lognormal	-0.49	0.07	[23]
*Transition probability of switching from second-line to third-line ART regimen*				
Constant in survival analysis for baseline hazard	Lognormal	-10.29	1.27	[23]
Age coefficient in survival analysis for baseline hazard	Lognormal	0.06	0.02	[23]
Ancillary parameter in Weibull distribution	Lognormal	0.01	0.14	[23]

SE: standard error; FSW: female sex worker; IDU: injecting drug user; and MSM: men who have sex with men.

**Table 2:** Resource costs parameters used in Markov model (THB as of 2009 value)

Parameters	Distribution	Mean	SE	References
Costs of prevention program				
Annual costs of existing prevention programs	Gamma	24		[27,28]
Cost of HIV vaccine per course	Gamma	3,500		[12]
Individual cost of community engagement	Gamma	937*		
Cost of HIV screening (ELISA) for vaccine acceptance	Gamma	125		[47]
Cost of pre-counselling for all vaccinations	Gamma	141		[47]
Cost of post-counselling for vaccine acceptance	Gamma	58		[47]
Costs of treatment program				
*Costs of asymptomatic treatment*				
Laboratory cost for asymptomatic patient	Gamma	8,155		[29]
Hospital service cost of asymptomatic patient	Gamma	2,502		[29]
OPD cost of asymptomatic patient	Gamma	2,502		[29]
*Costs of symptomatic treatment*				
Lab test cost for symptomatic patient	Gamma	8,931		[29]
Opportunity infection treatment cost of symptomatic patient	Gamma	4,739		[29]
Hospital service cost of symptomatic patient	Gamma	9,104		[29]
OPD cost of symptomatic patient	Gamma	2,502		[29]
IPD cost of symptomatic patient	Gamma	6,227		[29]
*Costs of AIDS treatment*				
Opportunity infection treatment cost of AIDS patient	Gamma	4,739		[29]
Hospital service cost of AIDS patient	Gamma	9,104		[29]
OPD cost of AIDS patient	Gamma	2,502		[29]
IPD cost of AIDS patient	Gamma	6,227		[29]
Annual drug costs of the first-line ART regimens (mg):	Gamma	8,184^†^	1,858^†^	
1. d4T(30)+3TC(150)+NVP(200) or				
2. d4T(30) + 3TC(150) + EFV (600) or				
3. AZT(100/200/250/300)+3TC(150)+NVP(200) or				
4. AZT(100/200/300)+3TC(150)+EFV(600)				
Annual drug costs of the second-line ART regimens (mg):	Gamma	32,478^†^	5,772^†^	
1. ddI(250)+3TC(150)+NVP(200) or				
2. ddI(250)+3TC(150)+EFV(600) or				
3. TDF(300)+3TC(150)+NVP(200) or				
4. TDF(300)+3TC(150/300)+EFV(600)				
Annual drug costs of the third-line ART regimens (mg):	Gamma	15,682^†^	2,080^†^	
1. AZT(100/200/300)+3TC(150)+Boosted PIs^‡ ^or				
2. d4T(30)+3TC(150)+Boosted PIs^‡ ^or				
3. TDF(300)+3TC(150)+Boosted PIs^‡ ^or				
4. ddI(250)+3TC(150)+Boosted PIs^‡ ^or				
5. AZT(100/200/300)+ddI(250)+Boosted PIs^‡ ^or				
6. AZT(100/200/300)+TDF(300)+Boosted PIs^‡ ^or				
7. AZT(100/200/300)+3TC(150)+TDF(300)+Boosted PIs^‡^				
Annual costs of lab test of first-line ART regimen in the first year	Gamma	7,671		[48]
Annual costs of lab test of first-line ART regimen in subsequence years	Gamma	4,210		[48]
Annual costs of lab test of the second-line ART regimen	Gamma	4,140		[48]
Annual costs of lab test of the third-line ART regimen	Gamma	4,163		[48]

SE: standard error; THB: Thai baht; OPD: outpatient department; IPD: inpatient department; ART: antiretroviral treatment; d4T: stavudine; 3TC: lamivudine; NVP: nevirapine; EFV: efavarenze; AZT; zidovudine; TDF: tenofovir; ddI: dianosine; and PIs: protease inhibitors.

*Nakorn Premsri, National Vaccine Committee Office, Department of Disease Control, Ministry of Public Health, personal communication, August 25, 2009.

^†^Thanapat Laowahutanon, AIDS office, Bureau of Disease Management, National Health Security Office, personal communication, August 20, 2009.

^‡^Boosted PIs were recommended by National Health Security Office as follows: the first-line PIs regimen, LPV/r-- lopinavir (200 mg) + ritonavir(50 mg) or IDV/r-- indinavir (400 mg) + ritonavir (100 mg) and the second-line PIs regimen, ATV/r-- atazanavir (150 mg)+ ritonavir (100 mg)

**Table 3:** Input parameters (i.e. utility parameters and characteristics of HIV vaccine) used in Markov model

Parameters	Distribution	Mean	SE	References
Utility parameters				
Utility of HIV negative		1		
Utility of asymptomatic patients	Beta	0.86	0.01	[24]
Utility of symptomatic patients	Beta	0.80	0.01	[24]
Utility of AIDS patients	Beta	0.76	0.01	[24]
Characteristics of HIV vaccine				
Vaccine efficacy	Gamma	31%	13%	[11]
Increased incidences of HIV infection compared to baseline due to the change of risk behaviors	Gamma	20%*	20%*	
Duration of booster doses (year)	Gamma	10*	10*	

SE: standard error.

*Based on assumption

The study also performed a value of information analysis to determine the population expected value of perfect information (EVPI) and the partial EVPI to determine whether different values of a particular input parameter lead to different optimum decisions [11,34-36] (see Appendix) and if so, how much the expected loss under alternative optimum decisions varies given the scenario that the vaccine efficacy is 31.2% (95% confidence interval-CI, 1.1 to 51.2) as revealed in a recent vaccine trial in Thailand [11]. The vaccine cost was fixed at 210 THB with an 80% vaccine acceptance rate and a 10-year vaccine protection duration. Because the analysis of partial EVPI requires an explicit statement of the value of the ceiling ratio, this analysis applied the Thai WTP threshold at 100,000 THB [30].

## Results

### Cost-utility analysis

The results shown in Table 4 are average lifetime costs and the QALYs of an HIV vaccination program compared to existing prevention programs. The results are classified by risk group. The study found that for the general population, the vaccine was most cost-effective for 18 year olds with an ICER of approximately 157,000 THB per QALY. For high risk population groups, the vaccine was very cost-effective for IDUs and MSM due to higher effectiveness and lower costs compared with existing prevention programs. For other high risk groups, the vaccine was cost-ineffective.

**Table 4:** Incremental cost-effectiveness ratios (ICERs) of HIV vaccination program compared to existing prevention program, classified by risk group

	HIV vaccination program		Existing prevention programs		ICER
	
	Costs (THB)	QALY	Costs (THB)	QALY	THB per QALY gained*
**General population **					
aged 18 years old	12,900	25.73	5,490	25.68	157,000
**FSW **					
aged 29 years old	47,300	23.46	46,800	23.25	2,840
**IDU **					
aged 26 years old	53,900	13.03	62,400	12.61	Dominated^†^
**MSM **					
aged 26 years old	243,000	16.51	245,000	16.27	Dominated^†^
**Male conscript **					
aged 21 years old	11,400	23.80	4,570	23.78	326,000

ICER: incremental cost-effectiveness ratio; FSW: female sex worker; IDU: injecting drug user; MSM: men who have sex with men; THB: Thai baht as of 2009 value; and QALY: quality adjusted life year.

*ICERs are rounded up to nearest 1,000 THB.

^†^Negative ICER due to higher effectiveness and lower costs of HIV vaccination program compared with existing prevention programs.

### Uncertainty analysis

#### One-way sensitivity analysis

**Figure 3:**
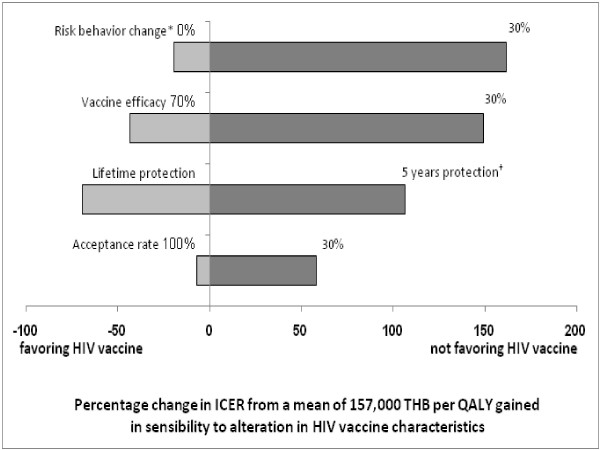
**One-way sensitivity analysis**. The diagram shows the sensitivity of ICER to hypothesis ranges of characteristics of the HIV vaccine. The numbers at each end of the bars indicate the most extreme values used in the sensitivity analysis. ICER: incremental cost-effectiveness ratio; THB: Thai baht as of 2009 value; and QALY: quality adjusted life year. *Percentage of risk behavior changes (0%-30%) due to vaccination (i.e. decreasing condom usage and increasing habit of needle sharing among injecting drug users) † The study assumed that each boosted revaccination required a full course and the revaccination was needed to maintain protection over a 30-year period.

A tornado diagram shown in Figure 3 illustrates the results of one-way sensitivity analysis, and indicates that the ICER per QALY gained was most sensitive to changes in risk behavior post-vaccination from 0% to 30%. This was followed by vaccine efficacy of between 30% and 70%, and the duration of protection in a range of from 5 years to a lifetime period, while the altering of acceptance to vaccination ranges between 30% and 100% had the least influence on the changing of the ICER.

Using the one-way sensitivity results, only the significant parameters (i.e. risk behavior change, vaccine efficacy, and duration of protection) were used to identify the maximum costs of the vaccine shown in Table 5. The highest costs of the vaccine that is still cost-effective under the Thai healthcare setting was for the scenario where the vaccine has 70% efficacy with lifetime protection; no changes occur in risk behavior post-vaccination; and the vaccine is provided to MSMs. Overall, the vaccine has the best cost-effectiveness when being provided to MSMs, followed by IDUs, FSWs, the general population aged 18 years old, male military conscripts, and the general population aged 30 years old.

**Table 5:** The results of threshold analysis present the HIV vaccine prices being cost-effective, classified by risk group

Vaccine prices (THB) being cost-effective at a WTP threshold 100,000 THB per QALY gained									
**Duration of protection**	**Lifetime**			**10 years 3 boosters with full course**^**†**^			**5 years 6 boosters with full course**^**†**^		

**Vaccine efficacy**	**30%**	**50%**	**70%**	**30%**	**50%**	**70%**	**30%**	**50%**	**70%**

Risk behavior changed*									

*General population aged 18 years old*									
Unchanged	4,400	7,900	12,000	1,100	2,700	4,300	-	730	1,600
Increased 10%	2,400	6,500	11,000	210	2,000	3,900	-	380	1,400
Increased 20%	-	4,500	9,600	-	1,100	3,400	-	-	1,100
Increased 30%	-	2,000	8,100	-	23	2,700	-	-	760
*General population aged 30 years old*									
Unchanged	-	410	1,100	-	-	-	-	-	-
Increased 10%	-	180	960	-	-	-	-	-	-
Increased 20%	-	-	780	-	-	-	-	-	-
Increased 30%	-	-	550	-	-	-	-	-	-
*FSW aged 29 years old*									
Unchanged	52,000	85,000	120,000	23,000	38,000	54,000	12,000	20,000	29,000
Increased 10%	-	37,000	92,000	-	16,000	41,000	-	8,000	22,000
Increased 20%	-	-	59,000	-	-	26,000	-	-	14,000
Increased 30%	-	-	22,000	-	-	9,500	-	-	4,500
*IDU aged 26 years old*									
Unchanged	57,000	96,000	140,000	35,000	58,000	82,000	21,000	34,000	48,000
Increased 10%	22,000	69,000	120,000	14,000	42,000	73,000	7,600	25,000	43,000
Increased 20%	-	45,000	110,000	-	28,000	64,000	-	16,000	38,000
Increased 30%	-	24,000	93,000	-	15,000	56,000	-	8,400	33,000
*MSM aged 26 years old*									
Unchanged	170,000	310,000	500,000	100,000	170,000	250,000	59,000	98,000	140,000
Increased 10%	-	40,000	260,000	-	24,000	140,000	-	14,000	82,000
Increased 20%	-	-	31,000	-	-	19,000	-	-	11,000
Increased 30%	-	-	-	-	-	-	-	-	-
*Military male conscript aged 21 years old*									
Unchanged	2,200	5,500	8,900	150	1,600	3,100	-	160	1,000
Increased 10%	-	3,100	7,500	-	560	2,500	-	-	650
Increased 20%	-	320	5,800	-	-	1,800	-	-	240
Increased 30%	-	-	3,800	-	-	850	-	-	-

THB: Thai baht as of 2009 value; WTP: willingness to pay; QALY: quality adjusted life year; FSW: female sex worker; IDU: injecting drug user; MSM: men who have sex with men.

"-" HIV vaccine would not be cost-effective.

*Risk behavior changed due to vaccination (i.e. decrease in condom use and increase in needle sharing among injecting drug users)

^†^The study assumed that each boosted revaccination required a full course and the revaccination was needed to maintain protection over a 30-year period.

In contrast, in certain cases it has been found that providing the vaccine free of charge to some particular groups is still cost-ineffective compared to the existing prevention programs. These cases are:

(1) where the vaccine has a range of 30% to 70% efficacy with 5-year and 10-year protection, and 30% efficacy with lifetime protection provided for the general population aged 30 years old.

(2) where the vaccine has 30% efficacy with 5-year protection, and no changed risk behavior post-vaccination provided for the general population aged 18-30 years old and male military conscripts.

(3) where the vaccine has 30% efficacy with all durations of protection, and changed risk behavior post-vaccination of 10% provided for FSWs and MSMs, and changed risk behavior post-vaccination of 20% for the general population aged 18 years old, and IDUs.

(4) where the vaccine has 50% efficacy with all durations of protection, and changed risk behavior post-vaccination of 20% provided for the general population aged 18 years old, FSWs, and MSMs.

#### Probabilistic sensitivity analysis

**Figure 4:**
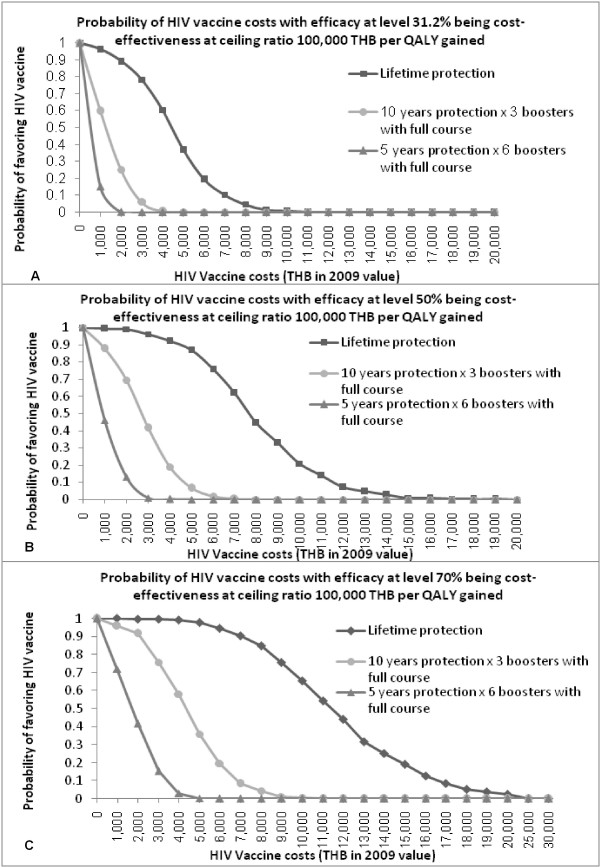
**Probabilistic sensitivity analysis**. For probabilistic sensitivity analysis in the base case (i.e., general population aged 18 years old, acceptance rate of 80%, and unchanged risk behaviors), these graphs demonstrate the probabilities of the HIV vaccine cost for each duration of protection and each vaccine efficacy level of (A) 30%, (B) 50%, and (C) 70% being cost-effective at the WTP threshold of 100,000 THB per QALY gained. ICER: incremental cost-effectiveness ratio; THB: Thai baht as of 2009 value; and QALY: quality adjusted life year.

Figure 4 illustrates the results of PSA in the form of graphs showing probabilities of different vaccine costs and the duration of protection being cost-effective at the Thai WTP threshold of 100,000 THB per QALY gained. The results show that when the efficacy is at 30% (Figure 4A), 50% (Figure 4B), and 70% (Figure 4C) with lifetime protection, the maximum cost could be defined as 4,000 THB, 7,000 THB, and 10,000 THB, respectively, with the probabilities of being cost-effective set at 60%, 62%, and 65%, respectively. For 10-year protection, the costs of vaccine efficacy of 30%, 50%, and 70% would be decreased to 1,000 THB, 2,000 THB, and 4,000 THB, respectively, with the probabilities of being cost-effective standing at 60%, 70%, and 58%, respectively. HIV vaccination with a 5-year duration of protection with 30% and 50% efficacy might not be cost-effective, while with 70% efficacy combined with a vaccine cost of 1,000 THB would provide a probability of cost-effectiveness of 72%.

#### Value of information analysis

**Figure 5:**
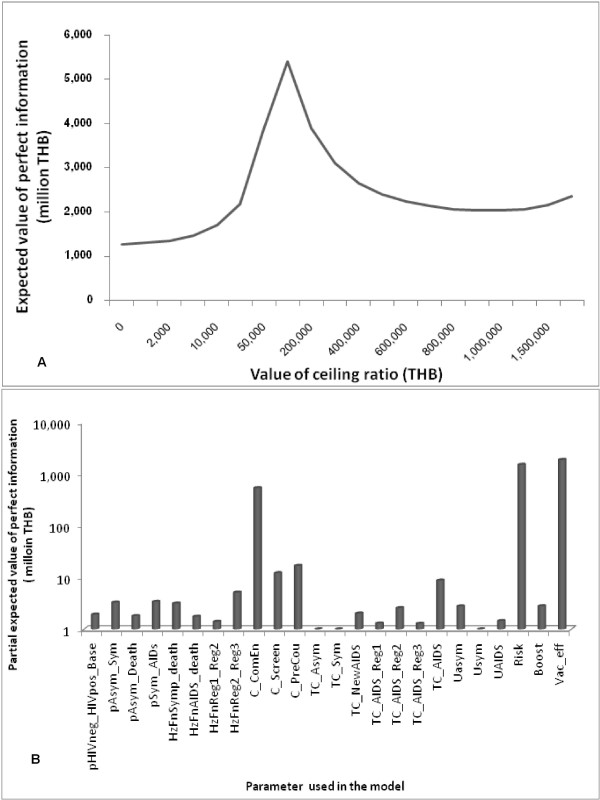
**Expected value of perfect information**. (A) Expected value of perfect population information for a model using the input parameters of general population aged 18 years, and using characteristics of vaccine hypothesis as follows: 31.2% of vaccine efficacy, 210 THB vaccine costs, 80% acceptance rate, 10-year protection, and 3 boosters with full courses. THB: Thai baht as of 2009 value. (B) Partial expected value of perfect information of parameters used in the model at a ceiling ratio of 100,000 THB per QALY gained for the provision of a HIV vaccination program for the general population aged 18 years and using characteristics of vaccine hypothesis as follows: 31.2% vaccine efficacy, 210 THB vaccine costs, 80% acceptance rate, 10-year protection, and 3 boosters with full courses. THB: Thai baht as of 2009 value; QALY: quality adjusted life year; pHIVneg_HIVpos_Base: the probability of HIV infection among the general population; pAsym_Sym: the probability of transition from HIV infection with asymptomatic state to a symptomatic state; pAsym_Death: the probability of transition from HIV infection with asymptomatic state to death; pSym_AIDS: the probability of transition from HIV infection with symptomatic state to AIDS state; HzFnSymp_death: the probability of death from HIV infection with symptomatic state analyzed from parametric survival analysis; HzFnAIDS_death: the probability of death during AIDS state analyzed from parametric survival analysis; HzFnReg1_Reg2: the probability of switching ART first regimens to second regimens among AIDS patients analyzed from parametric survival analysis; HzFnReg2_Reg3: the probability of switching ART second regimens to third regimens among AIDS patients analyzed from parametric survival analysis; C_ComEn: individual cost of community engagement; C_Screen: HIV screening cost; C_PreCou: cost of pre-counseling; TC_Asymp: total treatment cost of HIV infection in asymptomatic state; TC_Sym; total treatment cost of HIV infection in symptomatic state; TC_NewAIDS: total treatment cost of new AIDS patient; TC_AIDS_Reg1: total cost of treatment with the first ART regimens of AIDS patient; TC_AIDS_Reg2: total cost of treatment with the second ART regimens of AIDS patient; TC_AIDS_Reg3: total cost of treatment with the third ART regimens of AIDS patient; TC_AIDS: total cost of other treatment of AIDS patient; Uasym: utility weight of HIV infection with asymptomatic patient; Usym: utility weight of HIV infection with symptomatic patient; UAIDS utility weight of AIDS patient; Risk: percentage of change in the risk behavior post-vaccination; Boost: the duration of vaccine protection; and Vac_eff: vaccine efficacy

Figure 5A presents the population EVPI, i.e. the expected loss due to making a wrong decision at different ceiling ratios as a result of uncertainty in the overall parameters used in the model. The population EVPI for a 5-year period was analyzed in a hypothetical situation with the provision of a HIV vaccination program for the general population aged 18 years old with the hypothetical characteristics of the vaccine as follows: (1) an efficacy of 31.2% based on prime-boost efficacy trials, (2) a vaccine cost of 210 THB based on threshold analysis, (3) an acceptance of vaccination rate of 80%, and (4) 10-year protection and 3 boosters with full courses. The EVPI was highest at a ceiling ratio of 100,000 THB per QALY gained to be 5,400 million THB.

Since the results of population EVPI found the maximum value of the ceiling ratio to be at 100,000 THB, this ratio was taken for the partial EVPI analysis to examine the relative importance of each parameter used in the model. The results shown in Figure 5B present the estimated cost and the priority of further research to ascertain the perfect information of each parameter. The most important input parameters were vaccine efficacy and post-vaccination changes to risk behavior. The estimated value of partial EVPI of these 2 parameters stood at 1,900 million THB and 1,500 million THB, respectively. This was followed by the costs of the HIV vaccination program including community engagement, counselling, and screening for HIV infection where the total estimated partial EVPI was 570 million THB. The least important parameters were the probabilities of HIV infection, disease progression, the costs of HIV/AIDS treatment, and the utilities value of each HIV/AIDS state.

## Discussion

This is the first cost-utility study comparing a combination of HIV vaccination and existing HIV prevention programs with existing HIV prevention programs without HIV vaccination in both the general population and high-risk population groups. This study shows that providing a HIV vaccine is more cost-effective for high-risk groups than for the general population if the vaccine recipients do not change their risk behaviour post-vaccination. The changing of risk behavior after vaccination is one of the major parameters influencing the effectiveness and cost-utility of the vaccine, followed by vaccine efficacy, and the duration of protection. Therefore, adoption of the HIV vaccine with partial protection (30%-70%) into the healthcare system should be seriously considered alongside the potential change of risk behavior among the vaccinated population. In future clinical trials of the HIV vaccine these parameters need to be closely monitored. This study also helps inform decision makers of the maximum costs of HIV vaccination at which the vaccine would likely be cost-effective considering the Thai ceiling threshold of 100,000 THB per QALY gained [30].

Two economic evaluations previously published in international journals were identified [9,10]. One focuses on the use of a HIV vaccine with 30% efficacy in infants in Sub-Saharan Africa, while the other considers a HIV vaccine with 60% efficacy for 10-year-old children in Thailand. Both studies reveal that the HIV vaccine is cost-effective compared to non vaccination. Similarly to the results of this study, these two published papers also indicate that the cost-utility of a HIV vaccine depends very much on baseline HIV incidences and vaccine efficacy, although they did not consider the change of risk behavior. It is interesting to note that the results of these two papers did not favor the vaccine as much as the findings of this study because the major benefit of childhood HIV vaccination would not be observed until twenty-five years in the future.

Thailand experienced the use of economic evaluation for decision making regarding the adoption of new HIV prevention interventions. The first relates to the use of ART for the prevention of vertical HIV transmission or PMTCT [37,38]. In 2004, economic evidence supported the use of a combination of zidovudine (AZT) + nevirapine (NVP), and in 2010, the national policy was changed again when the economic analysis supported the use of a combination of AZT+ lamivudine (3TC) + lopinavir/ritonavir (LPV/r). The second policy change included the introduction of provider-initiated HIV counseling and testing instead of voluntary HIV counseling and testing. All of these interventions demonstrated that they yield a QALY at a cost that is below the Thai ceiling threshold [39]. This study is in line with the results from a household survey in Thailand which found that, when having sexual relations with partners who are not spouses, condom use among the HIV vaccinated was likely to be lower than among those not vaccinated [14]. Similarly, Newman et al. [40] reported the intentions of increasing post-vaccination risk behavior, including decreased condom use and an increased number of partners among the MSM population in Thailand if they were vaccinated with the HIV vaccine. Two studies [15,16] constructed epidemiological models and predicted that the introduction of a low efficacious HIV vaccine could worsen the HIV epidemic if increased risk behavior after vaccination was observed. As a result, introduction of the vaccine needs to be combined with other effective measures to prevent and monitor risk behaviors that may be changed post-vaccination, especially for a vaccine with only partial efficacy.

The findings of this study should be considered carefully. Firstly, this study did not account for the potential benefits of prolonged disease progression among vaccinated population groups who were subsequently infected by the virus [10]. However, the recent HIV vaccine trial in Thailand reported no difference in terms of viral load and CD4+ T-cell counts between vaccinated and non-vaccinated groups [11]. Secondly, although this study assumed a very high acceptance rate of HIV vaccination (80%), the model did not take into account the potential benefit of herd immunity. This could lead to an underestimation of the efficacy and cost-effectiveness of the vaccine. Thirdly, since the vaccine is not available in the market, the assumptions of vaccine characteristics needs to be derived from expert consultations in order to define the possible range of those parameters. However, this study extensively explored parameter uncertainty and provided valuable information using a new approach - EVPI analysis - to prioritize model parameters for future fine tuning. Fourthly, even though the societal perspective is recommended in the current Health Technology Assessment Guidelines in Thailand [41], this study adopted a government perspective in the analysis. This is because the direct medical care costs are likely to be a major cost component. A future study should be conducted using the societal viewpoint in which the increased economic productivity of individuals whose infection has been averted is counted. This would make the vaccine more preferable as empirical evidence indicated that HIV/AIDS significantly affected household economies [42]. Fifthly, this study did not consider other factors that are relevant to policy decisions regarding health resource allocation in Thailand. These include affordability, equity and politics, as well as social and cultural dimensions [43-45]. Finally, although this study offers a useful and comprehensive framework for evaluating the cost-effectiveness of HIV vaccination, all input parameters used in the analysis are mostly relevant to the Thai context and only a government perspective was used; therefore, applying these findings elsewhere should be done with caution.

## Conclusions

Because a HIV vaccine is not yet available in the market, this study was conducted using several assumptions including vaccine efficacy, duration of protection, and the change of risk behavior after vaccination. These assumptions were made with the careful consideration and involvement of various partners. This was to help with transparency, to encourage participation, and to help with the acceptance of this research. This approach differentiated this study from other economic evaluations and can be applied for the economic evaluation of other health interventions not available in healthcare systems. This kind of study can be very useful and important not only for researchers conducting future HIV vaccine research but also for policy decision makers who, in the future, will consider vaccine adoption in Thailand.

## Competing interests

The authors declare that they have no competing interests.

## Authors' contributions

PL performed the research, analyzed data, and drafted the manuscript. YT performed the research and drafted the manuscript. PW and CA participated in the analysis. NP, CN and WP provided the clinical data. VT participated in its design. All authors read and approved the final manuscript.

## Appendix

### Expected value of perfect information

The overall expected value of perfect information (EVPI) is the difference between the expected net benefit of the optimal strategy given perfect information, which can be written as:

and the expected net benefit of strategy that would be adopted given current (imperfect) information, which can be presented as:

The formula can be shown as follows:

where θ is the set of parameters for the model, which were assigned prior probability distributions; t is the set of possible decisions or strategies; and NB (t, θ) is the function of net benefit for decision t and parameters θ.

This is presented in terms of THB per patient, and then to the given population EVPI. The overall EVPI was multiplied by the proportion of the general population aged 18 and projected by the Thai Office of the National Economic and Social Development Board over a 5-year period. This was assumed to be the operating period of the HIV vaccination program in Thailand with a discounted rate of 3% per annum.

To quantify the value of receiving further information on the chosen parameters, partial EVPI is the difference between the expected value of a decision made with perfect information about a particular vector of the parameters (θ) and the current optimal decision.

With perfect information, θ_i _is the known vector of the parameters of interest θ; then the expected net benefit of a decision made would now be found by averaging over the uncertainty in θ that remains once we know θ_i _and then by selecting the optimal treatment that provides maximum expected net benefit, and can be written as:

At this stage, we do not have perfect information on θ_i_, so the expected value of any decision made with perfect information about θ_i _is found by averaging the uncertain ranges of the parameters θ_i _and can be presented as:

The additional value of collecting perfect information on a subset θt of uncertain model parameters is therefore given by the following equation:

The analysis of partial EVPI requires an explicit statement of the value of the ceiling ratio; therefore, this analysis applied the Thai WTP threshold at 100,000 THB.

## Pre-publication history

The pre-publication history for this paper can be accessed here:

http://www.biomedcentral.com/1471-2458/11/534/prepub
